# Perspective on patient-centered communication: a focus group study investigating the experiences and needs of nursing professionals

**DOI:** 10.1186/s12912-024-02487-7

**Published:** 2024-11-12

**Authors:** Kendra Mielke, Wiebke Frerichs, Katja Cöllen, Anja Lindig, Martin Härter, Isabelle Scholl

**Affiliations:** Department of Medical Psychology, University Medical Center Hamburg-Eppendorf, Martinistraße 52, 20246 Hamburg, Germany

**Keywords:** Nurses, Nursing professionals, Patient-centered communication, Communication skills training, Focus group interviews

## Abstract

**Background:**

Delivering high quality care tailored to patients’ needs necessitate patient-centered communication. High physical and mental workload, as well as organizational barriers, contribute to challenges nurses face in patient-centered communication. Participation in a communication skills training can help nurses to improve their patient-centered communication skills. Thus, the aim of this study was to investigate the experiences of nursing professionals in patient-centered communication and delineate the requisite content for a communication skills training.

**Methods:**

We conducted focus group interviews with nursing professionals working at an academic medical center in Germany. The focus group interviews were audio-recorded and transcribed verbatim. Data analysis was performed using Kuckartz’s qualitative content analysis.

**Results:**

31 nursing professionals from diverse medical disciplines (e.g., pediatrics, obstetrics, cardiology, neurology, oncology) participated in the study, unveiling a spectrum of communication experiences, including organizational and system-related challenges, constraints induced by the COVID-19 pandemic, management of personal emotions, and communication challenges with patients and relatives. They also identified aspects they experienced as beneficial for patient-centered communication (e.g., allocating sufficient time, being authentic, providing clear information). Furthermore, participants identified specific aspects, which should be included in a patient-centered communication skills training.

**Conclusion:**

The findings suggests that not only nurses from oncology and intensive care, but also from other medical disciplines, experience significant communication challenges with patients and relatives. Applying patient-centered aspects of communication was considered beneficial for effective communication. The needs identified through participants’ experiences (e.g., core communication skills and strategies, handling escalating situations, and discussing serious illness, death and dying) informed the development of a patient-centered communication skills training specifically tailored for nursing professionals across diverse medical disinclines in Germany.

**Supplementary Information:**

The online version contains supplementary material available at 10.1186/s12912-024-02487-7.

## Background

Patient-centered communication is crucial to high-quality care, influencing positive health outcomes of patients (e.g., health-related quality of life, physical and emotional well-being), patient-healthcare professional relationship [1], preventing sentinel and adverse events (e.g., patient safety) [2], and alleviating distress of nursing professionals [3]. Patient-centered care involves considering patients’ needs and preferences, and treating them with respect and empathy [4]. To achieve patient-centered communication, healthcare professionals should employ various verbal (e.g., providing tailored explanation, exploring psych-social context, offering reassurance) and non-verbal techniques (e.g., eye contact, nodding for understanding) [1, 4].

In clinical settings, nursing professionals manage multifaceted tasks, including physical, psychological, and social support for patients, offering information to relatives as well as handling administrative tasks [5]. To navigate these responsibilities, adequate professional preparation and training is essential [5]. However, nurses face increasing physical and mental workload, contributing to high sickness rates and reduced work capacity [6, 7].

The listed challenges contribute to nurses’ difficulties communicating in a patient-centered way [8]. International studies have examined communication experiences of nurses, often in specialized areas like oncology and intensive care. Yoo et al. [9] investigated communication challenges of Korean intensive care nurses. Participants face communication difficulties resulting from urgent cases and lack of experiences. They also reported negative experiences leading to doubts about their profession [9]. An US-based study among surgical intensive care nurses identified communication barriers, particularly regarding prognosis of the illness and end-of-life care [10]. In Hong Kong, Chan et al. [11] interviewed cancer care nurses, who reported prioritizing patients’ physical needs over psychosocial needs due to time constrains. These nurses also emphasized the importance of dealing with emotional challenges and the significance of improving communication skills through experiences, role modelling, and trainings [11]. The generalizability of findings from international studies to the German hospital nursing context remains unclear. Career options and associated responsibilities in German nursing care differ significantly from those in countries such as United States, Netherlands, Australia or United Kingdom [12, 13]. The proportion of nurses with an academic degree is low, and their roles do not substantially differ from those with a more common vocational training in nursing [13]. Additionally, the implementation of nurse practitioners (NA) and advanced practice nurses (APN) is limited in Germany, with restricted opportunities to perform advanced clinical activities (i.e., prescribing medications, treatment decisions, ordering medical tests) [12].

Research suggests that nurses’ patient-centered communication skills can be fostered through participating in a communication skills training (CST) [14–16]. While the previous mentioned studies identified communication challenges, results did not contribute to specific CST development. Only a limited number of international studies developed and evaluated a CST for nursing professionals through a preliminary assessment of their experiences and needs [15, 17].To our knowledge, there are currently no CSTs for nursing professionals from diverse clinical backgrounds in Germany that have proactively assessed communication experiences and resulting needs for development. Existing CSTs vary in quality and quantity [18], are often specific to certain medical disciplines (e.g., oncology), or have not been developed based on stakeholders’ needs and preferences [19]. Fernandez et al. [20] suggest that interventions can be more effective if target groups’ needs and preferences are identified beforehand. Therefore, our objective was to conduct a qualitative study using focus group interviews among nursing professionals in Germany before developing and evaluating a patient-centered CST.

## Methods

### Aim

The aims of this study were to explore nursing professionals’ (1) experiences with patient-centered communication as well as (2) their needs regarding the content of a future communication skills training.

### Design

We conducted a qualitative study using focus group interviews. Focus groups are designed to gather and analyze diverse opinions, experiences, and ideas from participants on a specific topic. The objective is to explore various perspectives rather than achieve consensus [21]. Therefore, this method is often used for the development of tailored interventions in health research [22]. The study was guided by the Consolidated criteria for Reporting Qualitative Research (COREQ statement) [23]. The COREQ checklist can be found in Appendix file 1.

### Study setting and recruitment

This study was part of a needs assessment conducted within the KOMPAT-study (German: KOMmunikation in der PATient: innenorientierten Pflege; English: Communication within patient-centered nursing care), which aims to develop and evaluate a patient-centered CST [24].

The focus group interviews were conducted at the University Medical Center Hamburg-Eppendorf (UKE), an academic medical center in northern Germany. We invited ward managers to disseminate digital and printed information about the focus group interviews to their staff at the end of 2021. Following this, reminder emails were sent to the ward managers, along with an offer to present the study during their staff meetings. Additionally, we published an invitation in the medical center’s weekly newsletter for employees including our contact details.

To ensure a sample with diverse knowledge and experience relevant to our research interests, we employed purposive sampling [25]. Based on literature and in order to facilitate discussion and maintain manageable group dynamics [22], we opted to conduct five focus group interviews with a maximum of 8 participants per group a priori. Additionally, ongoing monitoring was conducted during data collection to assess the extent to which new content was emerging (i.e., reaching data saturation) [26]. Eligible participants were informed about data protection and required to provide consent by signing a consent form beforehand. Participants received education credit points but did not get a monetary compensation for expenses as involvement in the focus group interview occurred during their working hours.

### Inclusion and/or exclusion criteria

Participants needed to be certified nurses or medical assistants (for better readability, we will refer to both professions as “nursing professionals”) with completed professional training, whose daily responsibilities include direct communication with patients. In Germany, medical assistants primarily work in outpatient facilities, performing administrative tasks and basic medical procedures such as blood sampling, X-rays, and injections. However, they are also engaged in both inpatient and outpatient facilities within hospitals. Their responsibilities vary by working area and encompass administrative functions (e.g., managing admissions and discharges, coordinating appointments), assistance roles (e.g., supporting physicians during examinations, documenting treatments and findings), and patient care activities (e.g., providing information, organizing follow-up treatment). Consequently, they were included in this study due to their significant involvement in patient communication, which is comparable to that of nurses. To participate in the study, nursing professionals had to be employed at one of the UKE’s departments. Further, they were excluded if they held a management position.

### Data collection

All focus group interviews were conducted between January and March 2022 in a seminar room at the research teams’ institute. Each focus group interview was audio-recorded. Two members of the research team (WF together with KC or KM) moderated all five focus group interviews (for researcher characteristics see section Rigor and reflexivity).

To simplify transcription, a student assistant took notes during each focus group interview. Participants’ demographic data were collected through a brief questionnaire. No relationship was established between participants and interviewers prior to this study. Participants did not receive the transcripts for comments and/or corrections, nor were they asked to comment the results.

The interview guide was developed and discussed with experts in conducting qualitative research (AL, IS, MH) as well as one nursing professional, who was not part of the research team nor participated in one of the focus group interviews (see Appendix file 2). During the focus group interviews, moderators first introduced themselves (e.g., name, background, experiences with research topic), then participants introduced themselves briefly. Next, the moderators provided an overview of the study’s background. The following themes were covered: (a) participants’ challenges in communicating with patients and strategies for addressing these challenges; (b) situations, in which communication with patients was effective; (c) participants were asked to write down up to three aspects on small cards that should be included in a CST. The moderators subsequently organized and clustered the cards and facilitated a group discussion based on the results.

### Data analysis

For this study, we analysed only responses related to nursing professionals’ experiences when communicating with patients as well as their needs regarding the content of a CST. Therefore, responses regarding participants’ previous experiences with communication skills trainings and organizational aspects for training development were not analyzed.

First, audio files were transcribed using the software F4 transcript (version 4.2, dr.dresing & pehl GmbH, Marburg, Germany), following the transcription rules of Dresing & Pehl [27]. Personal data was anonymized during transcription. We conducted data analysis via qualitative content analysis [28] using MAXQDA software (version 2020, VERBI GmbH, Berlin, Germany). Two team members (KC and KM) developed deductive categories based on the interview guide. Then KM and KC read all transcripts and each double coded 40% of the transcripts (2 of the 5 transcripts), developing gradually inductive categories, thus creating a preliminary coding scheme. This preliminary system was then discussed with WF and adapted accordingly. Subsequently, KC and KM divided the remaining three transcripts between them for analysis, using the preliminary category system and made shared adjustments once new sub-categories emerged. During that step, WF was consulted if necessary. KM employed the preliminary category system to code the entire material again, making final adjustments, resulting in the final category system.

### Ethical considerations

This study was approved by the Local Psychological Ethical Committee of the Center for Psychosocial Medicine at the University Medical Center Hamburg-Eppendorf (approval number LPEK-0367). This study was carried out in accordance to the latest version of the Helsinki Declaration of the World Medical Association. Principles of good scientific practice was respected. Requirements for data protection were met. Participants gave written informed consent to participation and audio recording of the focus group interview. Participation in the study was voluntary.

### Rigor and reflexivity

All researchers involved in this study had previous experiences in conducting qualitative research. KM is a female health scientist (M.A.), trained physical therapist and a doctoral researcher. WF is a female health scientist (M.Sc), international physical therapist (B.Sc.), and experienced doctoral researcher. KC is a female psychologist (M.Sc.) and a trained nurse. AL is a female neuro-psychologist, trained psycho-oncologist, and post-doctoral researcher. MH and IS supervised the trial as principal investigators. MH is a male physician, psychologist, and professor for medical psychology. IS is a female psychologist, and professor for psycho-oncology. The research team has extensive experience in developing, evaluating and implementing various forms of CSTs, including undergraduate and postgraduate courses in medical school, continuing education courses and trainings within research settings [29].

## Results

### Demographic data

*N* = 46 nursing professionals showed interested to participate in one of the focus group interviews. Overall, *n* = 31 nurses participated in *n* = 5 focus group interviews. *N* = 15 nurses did not participate due to following reasons: exclusion due to management position (*n* = 5), had to work spontaneously (*n* = 2), sickness (*n* = 2), or unknown reasons (*n* = 6). Each focus group interview included five to seven participants and lasted between 80 and 85 min. 87% of the participants were female (*n* = 27), 39% younger than 30 years (*n* = 12) and 39% (*n* = 12) had over 20 years of professional experience as a nurse. Table 1 provides detailed participants’ characteristics.

**Table 1:** Focus group interviews participants’ characteristics (*n* = 31)

	*n* (%)
Gender	
Female	27 (87%)
Male	4 (13%)
Age	
< 30 years	12 (39%)
31–40 years	7 (23%)
41–50 years	6 (19%)
> 50 years	6 (19%)
Profession	
Nurse	24 (77%)
Pediatric nurse	4 (13%)
Medical assistant	2 (17%)
No answer	1 (3%)
Working area	
Inpatient service	17 (55%)
Outpatient service	11 (36%)
No answer	3 (10%)
Department	
Pediatrics & obstetrics	5 (16%)
Internal medicine	4 (13%)
Cardiology	4 (13%)
Neurology	4 (13%)
Oncology	3 (10%)
Emergency medicine	3 (10%)
Others	3 (10%)
Psychosocial medicine	2 (7%)
Surgical medicine	2 (7%)
Outpatient clinic	1 (3%)
Professional experience in nursing care	
< 5 years	6 (19%)
5–10 years	7 (23%)
11–20 years	6 (19%)
> 20 years	12 (39%)

### Participants’ experiences with patient-centered communication and resulting needs

Based on our interview guide (see Appendix file 2), two deductive main categories emerged from the data: [1] communication experiences and [2] needs for a communication skills training. Respective inductive sub-categories had been extracted and are presented in Table 2.

**Table 2:** Main and sub-categories

Main category: Communication experiences	Main category: Needs for a communication skills training
Sub-categories: • Communication challenges with patients • Communication challenges with relatives • Managing personal emotions • Organisational and system-related challenges • Constraints induced by COVID-19 pandemic • Effective patient-centered communication	Sub-categories: • Communication with demanding or aggressive patients • Communication concerning serious illness, death and dying • Core communication skills and strategies • Communication with relatives • Cultural aspects of communication

### Main category: communication experiences

#### Communication challenges with patients

Participating nurses highlighted their challenges in direct patient interactions. During discussions, participants reported that they regularly have to deal with angry or aggressive patients. One participant mentioned that verbal assaults often arise due to extended waiting times or restrictions on accompanying relatives. Additionally, challenging behavior resulting from alcohol or drug misuse were commonly cited (e.g., verbal aggression, solely prioritizing own needs). Moreover, nurses noted experiencing pressure due to patients’ attitudes, *“[…] I think there is a lot of pressure on us from patients and relatives. It’s precisely this idea […] ‘I’m an emergency*,* why do I have to wait now’. That is difficult.” (FG5P2.)*

Nursing professionals reported a lack of patients’ necessary adherence or understanding of potential implications a specific illness can have. They elaborated difficulties encountered in motivating patients to change certain behaviors or to engage them in specific care activities: *“Sometimes*,* they [the patients] try to discuss everything and expect everything to be done for them. So*,* it’s very*,* very difficult for us to motivate them […]*,* to engage in their own activities*,* to take their own actions. And that’s also very challenging when you sometimes feel like it’s more of a service hospital.” (FG3P2).*

Participants experienced also challenges with patients having physical impairments (e.g., hearing impairment) or psychological disorders (e.g., alcohol abuse, mental disabilities, dementia) and resulting consequences (e.g., reduced adherence, hallucination). A participating nurse shared her experience with patients experiencing hallucinations: *“There are patients who call the police because they believe they are being detained. It’s important to approach them empathetically*,* trying to communicate clearly that there is no danger […]*,* as this situation occurs frequently.”* (FG4P4).

Another challenge mentioned was communicating with patients facing language barriers, as this can be time-consuming: “[…] *as I have already indicated*,* if the language barrier is relatively high*,* then it takes an incredible amount of time.*” (FG5P6).

Next to language barriers, a nurse highlighted the challenge of addressing specific requirements associated with patients from diverse cultural backgrounds: *“[…] we allocate our beds based on emergency cases*,* so we may have men and women together if necessary and this can cause significant problems with certain faiths and ethnic groups”* (FG4P3).

Sexual harassment was identified as challenging, especially for young or unexperienced female nursing professionals. A participating nurse expressed her uncertainty in dealing with inappropriate language or behavior by asking *“[…] how can I set verbal boundaries?”* (FG5P3).

Discrimination and the use of offensive language can also cause problems. Some participants reported instances where discriminatory language was perceived as deliberate attempts to offend staff or as being humorously. One participant expressed the need for training on how to address discriminatory remarks: *“[…] it’s really helpful for many to have some sort of training on how to deal with it [discriminatory remarks]. How can I assert myself if it comes to that*,* especially since the issue of racism arises among patients on the ward at times?”* (FG4P4).

Addressing serious illness, death, or dying poses a significant challenge for nursing professionals. Participants highlighted their difficulties of responding to patients who have just received distressing news. Additionally, end-of-life communication was identified as a significant challenge. A nurse remarked that it is challenging “*[…] to find the right words*,* avoiding overwhelming [the patient] with technical knowledge*,* finding comforting words instead”* (FG 2P5).

Nursing professionals also acknowledged the challenges associated with patients’ fear of receiving a bad diagnosis or worries regarding unknown treatments and their potential side effects. One nurse emphasized the difficulty of remaining calm while providing appropriate support to the patient and described a situation where *“[…] the patient was incredibly scared because he felt threatened […]”* (FGP4).

#### Communication challenges with relatives

Many participants concurred that they struggle with high expectations of relatives, which can often complicate communication compared to interactions with patients. Some participants reported that maintaining professionalism is a challenge. They also found dealing with relatives to be quite exhausting: *„I think it is even more difficult [to deal] with relatives than with patients.” (FG1P4).*

#### Managing personal emotions

Participants reported that it is often difficult not to be offended by rude patients. It was seen as crucial to maintain a professional distance to prevent being overly affected by patients‘ sadness and depression. One participant mentioned the difficulty of continuing to work after a patient passed away. Participants emphasized the importance of not letting patients perceive their own stress or insecurity: *„I am that kind of person*,* I try to give 100%*,* even if the shift is tough. And you try not to let the patients notice that you are completely stressed. And that always made me sick*,* I was constantly catching colds […]” (FG1P2).*

#### Organizational and system-related challenges

Certain system-related barriers were seen as challenging for an effective patient-centered communication. Participants identified time pressure as a significant factor, exacerbated by concurrent tasks (e.g., addressing patient and relative inquiries, handling telephone calls, attending to patients with medical emergencies). Moreover, staff shortages, long patient waiting times, and a lack of privacy during consultations, contributed to additional stress experience.

Regarding challenges originating from organizational procedures, one participant mentioned the issue of premature discharges. This was supported by other participants, who explained that patients are often discharged before they are fully recovered and may lack adequate care upon returning home, which can lead to readmission just a few days later. Furthermore, inadequate information exchange between departments, uncertainty regarding responsibilities, and confusion surrounding upcoming treatments were cited as additional challenges: *„ […] that the information is not reaching the patient. As if there is a hole somewhere where it is not being passed on. And that regularly leads to problems for us.”* (FG5P5).

#### Constraints induced by COVID-19 pandemic

Since we conducted the focus group interviews during the COVID-19 pandemic, participants reported communication challenges arising from hygiene restrictions, such as the requirement to maintain distance whenever possible. Particularly challenging was the lack of facial expression caused by the necessity of wearing medical masks. Participants pointed out patients’ increased need for information due to concerns about current regulations and uncertainties surrounding COVID-19 pandemic. This circumstance was compounded by reduced staff availability (e.g., due to quarantine regulations), resulting in less time available to communicate with patients: *„ […] since corona*,* we have been working in pairs of two per shift - normally*,* we are four- there is no time to communicate at all.” (FG1P5).*

#### Effective patient-centered communication

Participants also discussed how their behavior can contribute to effective communication with patients and their relatives. Effective communication occurs when nurses focus on patients by acknowledging patients’ needs and reassuring them that they are being cared for. Additionally, nurses felt that questioning or mirroring patient’s behavior can help to de-escalate tense situations. *„ […] For me*,* communication is always good and positive when I really focus on the patient and one nothing else for that moment […].” (FG2P5).*

One nurse emphasized the importance of empathy in navigating various situations. Other participants considered a positive attitude towards patients to be important. Authenticity was also seen as beneficial for communication. Hence, having a diverse range of personalities within the team was considered useful, as patients respond differently depending on the nurse’s personality. Additionally, building relationships (e.g., by listening to patients’ private problems) was seen as a valuable aspect contributing to effective communication: *„I think it is kindness in general. I always try to put myself in the patient’s shoes like “I am the patient and I am in the waiting room.” (FG3P3).*

It was noted that structuring conversations and taking time to explain subsequent procedures or information enhances the effectiveness of communication. Additionally, allocating sufficient time for conversations was seen as beneficial as it supports a calm atmosphere and prevents long debates or escalations: *„ […] if it [the communication] is not on the fly*,* if we really […] make appointments with our patients for a certain time period and if you then structure yourself so that you can stick to the times [then communication works well].” (FG4P6).*

Explaining situations while being reliable was also considered beneficial. Moreover, offering patients different options whenever feasible and allowing them to make decisions was identified as a useful approach. Participants emphasized the importance of providing clear and understandable information: *„[…] I have made the experience that giving information constantly [helps patients]*,* but not too much information at once and rather short sentences.” (FG5P4).*

Participants shared that routine procedures contribute to a sense of security among patients. Simultaneously, this routine not only comforts patients but also enhances patients’ understanding for nurses and their working environment on the ward: *[…] Many people start to relax after two or three days. Because then they know us a bit*,* they know the place*,* they know roughly how thinks work here*,* and that has a very relaxing effect.” (FG3P2).*

### Main category: needs for a communication skills training

By exploring and clustering participants’ needs regarding the content for a CST, we identified various sub-categories. Terms enclosed in quotation marks represent those aspects written on cards by participants in response to the question about desired content for a CST (further explanation see section data collection).

#### Core communication skills and strategies

Participants frequently highlighted the need for general skills and strategies for patient-centered communication that should be included in a CST. Participants referred to communication skills and strategies for (i) recurring situations (i.e., ‘informed consent discussion aiming to provide structure to patients and relatives’, ‘knowledge transfer in an easy way’, ‘admission/discharge interview’), (ii) use of appropriate language (i.e., ‘open and closed questions’, ‘respectful, easy, understandable language’, ‘voice level’), and (iii) time & setting (i.e., ‘handling patients’ need for a conversation when time is limited’, ‘communication skills in stressful situations/when time is limited’, ‘enabling conditions for effective communication’). Additionally, nurses listed skills such as ‘showing empathy & being mindful’, ‘knowledge about different communication models’ (e.g., four-ears-model, nonviolent communication), and ‘non-verbal communication’ as essential content for a CST. Strategies for managing personal emotions were mentioned including ‘being authentic’, ‘staying calm’, ‘understanding personal boundaries’, ’self-observation’, and ‘resilience training’. Referring to these needs, one participant mentioned that *“[…] it is important to understand that it is not against me as a person*,* but […] to learn that I do not need to feel assaulted.”* (FG1P7).

#### Communication with demanding or aggressive patients

Participants identified various needs related to handling difficult situations, including ‘aggression’, ‘difficult patients’, ‘handling verbal violence’, ‘overbearing patients (especially with young employees)’, de-escalation’, and ‘non-violent de-escalation’. In subsequent discussions, one nurse emphasized the importance of de-escalation skills for self-protection, highlighting that aggressive or assaultive behavior is particularly challenging for young nursing professionals. Another nurse commented on patients being aggressive and the lack of competencies: “*Many colleagues do not know how to react. What should they do now?”* (FG1P3).

#### Communication concerning serious illness, death or dying

Participating nurses identified ‘off-limits’, ‘handling patient’s grief’, and ‘bereavement/deaths’ as desired content for a CST. They also stated ‘communication for motoric aphasia’. In the following discussion, nurses mentioned the lack of skills for communication in specific situations, such as *“[…] patients who cannot communicate at all because […] they are severely disabled […].”* (FG3P6).

#### Communication with relatives and cultural aspects of communication

Regarding communication with family members, participants mentioned following needs on the cards as important content for a CST: ‘communication with relatives/parents’, ‘handling conflicts between nursing professionals and relatives’, and ‘learning communication skills with relatives’. One participant also mentioned the need regarding ‘language barriers’ on one card, followed by a subsequent discussion leading to general aspects of cultural sensitivity and communication. For example, one nurse reported *“[…] on our ward*,* we often see relatives praying*,* and you enter the room for ward rounds […]*,* thinking ‘Well*,* I am interrupting them now. Otherwise*,* we are a hospital here. But it is their culture*,* they need to pray*,* and it is not possible elsewise.”* (FG1P6).

## Discussion

The present qualitative study explored nursing professionals’ experiences regarding patient-centered communication, along with participants’ needs concerning the content for a training enhancing patient-centered communication skills. The results revealed that interviewed nursing professionals face various challenges in communication with patients and relatives. Still, they expressed clear ideas about the essential elements for effective communication. In addition, all participants identified needs relevant for the development of a specific training.

In this study, reported communication challenges in daily nursing practice align with international qualitative research on communication in nursing practice. Previous studies consistently highlight challenges such as organizational procedures, resulting stress, and time constrains for nursing professionals [10, 11, 30]. Stress and time constrain can hinder the development of meaningful nurse-patient relationships [31] and lead to unfulfilled care tasks known as missed care [32]. Participants reported leaving communication with patients undone due to time limitations, which affects care quality [32]. Another mentioned challenge was managing personal emotions. Emotional involvement (e.g., close relationships with relatives, death of patient) contributes to nurses’ stress, anxiety, and burnout symptoms [33], although positive experiences potentially mitigate these symptoms [33]. Awareness and reflection on personal emotions can be considers as a perceptional skill. According to Denniston et al. [34] perceptional skills involve *“awareness of self and others and how that impacts communication (e.g.*,* thoughts*,* feelings*,* attitudes and biases)”.* Given the complex nature of communication in clinical settings, it is essential to incorporate not only technical skills (such as knowledge and behavior), but also perceptional skills. Although these skills are crucial for effective communication, most CSTs lack focus on this reflective aspect [34]. Our study participants noted that the already tensed working conditions for nursing professionals in Germany were intensified by the COVID-19 pandemic and its resulting restrictions. Recent research concluded that during COVID-19 pandemic, nursing professionals’ physical and mental exhaustion increased [35], while ethical challenges, like working despite own health risks, were prevalent [36]. Challenging communication with relatives was reported, echoing findings of difficulties due to urgency in intensive care unit [9], missed skills [10, 30, 37], and unrealistic expectations [37]. Additionally, participants reported challenges in communicating with aggressive and/or demanding patients, aligning with results from Banerjee et al. [30]. Cultural background and language barriers were mentioned as a reason for potential communication challenges, which is also consistent with prior studies [10, 30]. Additionally, our participants reported challenges when communication with patients about serious illness, death and dying, a challenge echoed in studies primarily conducted in oncology and intensive care [9, 10, 30, 37]. However, the present study included participants from various medical disciplines (i.e., pediatrics, gynecology, neurology, nephrology, and dermatology), demonstrating that nursing professionals across all disciplines encounter challenges in communicating with seriously ill patients and discussions about death and dying. Given the lack of research addressing these communication needs across multiple medical disciplines, further research is required.

Participants’ responses regarding effective communication aligned with dimensions outlined in the integrative model of patient-centeredness [4, 38], which are divided into principles, enablers, and activities. Participants mentioned aspects such as kindness, allocating sufficient time, and authenticity, corresponding to the dimension ‘essential characteristics of the clinician’, describing healthcare professionals’ attitudes such as empathy, respect, and tolerance. The mentioned aspect ‘focus on the patient’ aligns with the dimension ‘patient as a unique person’, emphasizing the recognition of patient’s individual needs, feelings, and values. The dimension ‘patient information’ highlights the importance of giving tailored and understandable information while considering patients’ needs and preferences, which corresponds to the mentioned importance of giving clear information Additionally, participants emphasized that effective communication involves routine procedures. This aspect aligns with the dimension ‘coordination and continuity of care’, highlighting the need for coordination across various clinical areas and a comprehensive understanding of patient data. Hence, we concluded that participants perceived communication as effective when the dimensions of the patient-centeredness model are applied.

Regarding the content for the CST, most needs mentioned were related to specific situations, which have already been discussed in the paragraph referring to the main category ‘Communication experiences’. A novel aspect that emerged was the need to enhance core communication skills and strategies. These findings resonate with the study conducted by Chan et al. [11], where nurses stressed the importance of ongoing training in communication skills with a practical focus. In nursing practice, communication with patients often occur unplanned and simultaneously with other, more technical care activities (i.e., administering medication, measuring blood pressure) [39]. To provide patient-centered communication, nursing professionals need to be aware of this dynamic and receive regular training to effectively apply communication skills and strategies, especially in unplanned situations [39].

### Strengths and limitations of the work

One strength of the study is the heterogeneity of participants’ work experiences and working area. We recruited participants from different departments and thus from different medical disciplines (e.g., oncology, cardiology, pediatrics, intensive care). There was also a wide range in terms of age and professional experience.

This study has some limitations. Due to the methodology of the present study, we only included nursing professionals working at one academic medical center. To what extent the present results also apply to nursing professionals from other hospitals cannot be inferred from this study. Study information for the focus group interviews were distributed by department and ward managers to the nurses. Although we emphasized that participation in the focus group interviews was voluntary, we cannot rule out the possibility that some nurses were encouraged by their managers to participate. Furthermore, we cannot guarantee that all nursing professionals received the study information due to the method of information dissemination.

## Conclusion

Nursing professionals face various challenges when communicating with patients and relatives, which are related to patients’ behavior and their individual burden of illness, relatives’ behavior, nurses’ emotional stress experience, and organizational constraints, hindering patient-centered communication. Many of the mentioned experiences align with findings from international studies. However, these previous studies primarily focused on oncology and intensive care nursing professionals. Despite differences in medical disciplines and professional experiences among participants in this study, their opinions regarding the CST’s content were similar. This indicates that nursing professionals in medical fields beyond oncology and intensive care face diverse communication challenges and should thus be provided with the opportunity to participate in a CST applying to all nursing professionals regardless of their setting or fields. Despite the communication challenges, nurses identified aspects that positively influence communication and foster patient-centeredness. When developing a CST, these positive aspects – such as showing kindness, being authentic and providing clear information - should be prioritized given that nursing professionals have firsthand experience with these aspects. This recognition could enhance their connection with these experiences, thereby serving as a foundation for acquiring new patient-centered communication skills.

The results of this qualitative study informed the development of a CST tailored to the needs of nursing professionals across various medical disciplines in Germany. While developing the CST, we focused on incorporating the aspects that the majority of study participants identified as required and necessary content (e.g., core communication skills and strategies, navigating communication in escalating situations, and communication about serious illness, death and dying). The CST is currently being conducted and evaluated using a randomized controlled study design [24].

## Electronic supplementary material

Below is the link to the electronic supplementary material.

Supplementary Material 1

Supplementary Material 2

## Data Availability

The datasets used and analyzed during the current study are not publicly available due to their close association with participants’ working area, making it difficult to sufficiently anonymize all personal data in all documents. However, the datasets are available from the corresponding author on reasonable request. The data set used in this study are in German language.

## Abbreviations

AL: Anja Lindig
APN: Advanced practice nurses
COREQ: Consolidated criteria for Reporting Qualitative Research
CST: Communication skills training
IS: Isabelle Scholl
KC: Katja Cöllen
KM: Kendra Mielke
MH: Martin Härter
NA: Nurse practitioners
UKE: University Medical Center Hamburg-Eppendorf
WF: Wiebke Frerichs
