# Supplementary material for: Perspective on patient-centered communication: a focus group study investigating the experiences and needs of nursing professionals
Source: BMC Nurs. 2024 Nov 12;23:822. doi: 10.1186/s12912-024-02487-7 (PMC11558982; doi:10.1186/s12912-024-02487-7)
Supplement: Supplementary file 1 — Supplementary Material 1 [file 12912_2024_2487_MOESM1_ESM.docx]

Interview guide: Focus group interviews

**A. Start**

- Introduction of the research team
- Provision of information about the study objectives
- Explanation of the procedure (duration, role of participants, informed consent, data protection, audio recording)

**B. Introduction of participants**

- Participants introduce themselves, stating their name, medical discipline, motivation to participate

**C. Communication experiences**

- Presentation of a PowerPoint slide explaining the term „communication“ in the clinical setting

1. „Which situation regarding communication with patients do you find particularly challenging in your daily work routine?“

2. „Referring to what you said before, how do you handle these challenging situations?

(„How do you handle these challenging situations? Where do you find support? Which person or specific technique helps you?“)

3. „In which situation does communication with patients work well?“

(„What helps you in these situations?“)

**D. Exercise**

4. „We would like you to write down your top three aspects that should be incorporate in a communication skills training.“

Discussion about aspects:

- „Which aspects are important for all medical disciplines?“
- „Do you feel any aspects are missing?“
